# Differential Privacy Preservation for Location Semantics

**DOI:** 10.3390/s23042121

**Published:** 2023-02-13

**Authors:** Liang Yan, Lei Li, Xuejiao Mu, Hao Wang, Xian Chen, Hyoseop Shin

**Affiliations:** 1 College of Computer Science and Technology, Chongqing University of Posts and Telecommunications, Chongqing 400065, China; 2 Chongqing Planning and Natural Resources Information Center, Chongqing 401147, China; 3 Key Laboratory of Tourism Multisource Data Perception and Decision, Ministry of Culture and Tourism, Chongqing 400065, China; 4 Data Science Laboratory, Konkuk University, Seoul 05029, Republic of Korea; 5 Division of Computer Science and Engineering, Konkuk University, Seoul 05029, Republic of Korea

**Keywords:** location-based services, location semantics, differential privacy, personalization

## Abstract

With the rapid development of intelligent mobile terminals and communication technologies, location-based services (LBSs) have become an essential part of users’ lives. LBS providers upload and share the collected users’ location data. The more commonly used methods for location privacy protection are differential privacy and its extensions. However, the semantic information about location, which is an integral part of the location data, often contains sensitive user information. Most existing research methods have failed to pay enough attention to protecting the semantic information in the location data. To remedy this problem, two different scenarios for location semantic privacy protection methods are proposed in this paper to address single-point and continuous location queries. Simulation experiments on real social location check-in datasets, and comparison of three different privacy protection mechanisms, show that our solution demonstrates good service quality and privacy protection considering location semantics.

## 1. Introduction

With the rapid development of the Internet and intelligent mobile terminals, location-based services (LBS) are being used more frequently. LBS can provide users with many services, such as location check-in, information pushing, and marketing pushing in the vicinity through location-based technology. The increase in the number of mobile phone manufacturers has dramatically reduced the price of mobile phones, brought smartphones into ordinary people’s lives, and accelerated the rapid development of LBS. The various applications based on LBS bring us considerable convenience. LBS can find locations close to home, including supermarkets, libraries, and training courses, based on the user’s location information. The rapid development of LBS has resulted in significant convenience for users and has been fully integrated into all areas.

The rapid development of LBS also creates new challenges. To make relevant pushes according to user preferences, LBS providers upload and share a large amount of collected user location information. However, the shared location data may involve some users’ sensitive information, which leads to the leakage of users’ information. Location information can reflect the user’s habits, such as the user’s home address, religion, interests, and the address of the company where the user works [1]. In real-world applications, if one wants to use location-based services (LBSs), one must upload their accurate location. However, the location data are sensitive for individuals since they can disclose an individual’s real-time position. Individuals do not want to upload their location data. Thus, there is a contradiction between location sharing and disclosing.

From the user’s point of view, privacy protection can be divided into semantic and spatiotemporal security. Spatiotemporal privacy protection mainly focuses on the user’s geographic location, based on using the current geographic location to determine the user’s nearby information, to thereby obtain the user’s personal information, including the user’s interests and health status. Location semantics indicates that the user is in a semantic range; the mining of the semantics can provide sensitive information related to the user, such as the semantic range of the user’s hospital, according to the inference that the user may be a patient, so the user’s personal information has been leaked. An attacker who knows a person’s location semantics (e.g., a specific restaurant or company) can launch an attack. Perturbing the precise latitude and longitude data does not protect an individual’s location privacy.

From the perspective of the overall architecture of the LBS system, the current user privacy leakage is generally divided into that related to internal attackers and external attackers. Internal attackers mainly operate through the relevant management personnel of the server by stealing information or leaking information, and external attackers steal information mainly through the user’s location information collected when using location services, based on which they estimate the actual location of the user. The attacker can use the obtained geographic location, the background knowledge of the user and the surrounding environment, and other information to make inferences. The more typical of these are semantic-based attacks [2] and area boundary attacks [3].

Numerous researchers have proposed many solutions to the location privacy leakage problems mentioned above. There are three main types of solutions: spatial anonymity-based, encryption-based, and location-distortion-based location privacy protection solutions. Spatial anonymity-based methods hide the region of location by anonymity algorithms. They do not publish the accurate sites but the area of the locations. Encryption-based techniques extend the idea of encryption to locations and mask the actual values of the latitude and longitude data. The published results are random values instead of accurate data. Regardless of their massive computing and storage consumption requirements, encrypted location data are not convenient for data mining. This is the biggest problem if encryption-based methods are used to protect individual location information. Location distortion-based methods hide the actual location value by perturbing the latitude and longitude data, and the uploaded results are the perturbed data. Due to the advantage of high-level data utility, perturbation-based methods have become popular for preserving an individual’s location privacy. State-of-the-art methods attempt to protect an individual’s location privacy by adding noise to the latitude and longitude data to perturb the accurate location.

Nonetheless, the added noise is always tiny to ensure good data availability, which leads to the issue that the location semantics may remain the same even if the latitude and longitude data are perturbed. In other words, the current methods cannot protect location semantics. Although some schemes have been proposed to address the location privacy problem, the following issues remain to be addressed:

(1) Low-level privacy degree. Current methods attempt to hide the actual value of the location, but location semantics is also sensitive information for individuals. It cannot provide enough privacy preservation even if the latitude and longitude data are hidden. The attacker can still know the location semantics. Current methods have the problem of a low-level privacy degree.
(2) Personalized protection. State-of-the-art schemes regard the locations equally. However, different areas have different sensitivity to individuals. For example, users are sensitive to their home and company addresses but do not care about the coffee shop or restaurant address. Thus, we should provide a personalized protection solution to achieve a better trade-off between utility and privacy.

The above two challenges mean that state-of-the-art methods need to be more appropriate for location semantics release. Thus, this paper presents a novel solution to address these issues. In terms of the first issue, we find that we can perturb the location data and search the corresponding nearby semantics. We can publish the nearby semantics around the user. Then, the semantics is protected. To address the second issue, we can calculate the sensitivities of different location semantics according to the visiting frequency. Then, we select the semantics according to the candidate’s sensitivity to publishing. In this case, we can protect an individual’s location semantics and provide personalized protection.

Inspired by these considerations, in this paper, we mainly focus on the theoretical basis of location privacy protection and investigate the privacy leakage problem caused by the semantic attack of the single-point location query service and the semantic inference attack of the trajectory continuous query service. Because the traditional differential privacy and anonymization schemes do not consider the semantic information of users, based on this, the two schemes are improved, and two corresponding privacy protection methods are proposed, respectively:

(1) We propose a Differential Privacy protection Algorithm for Location Semantics (DPALS) to defend against the semantic attack of a single-point location query service. The method uses “geographic indistinguishability” to generate multiple perturbed locations and considers the semantic rank of the location. Based on this idea, we construct an anonymous set of location semantics conforming to the semantic privacy rank, and design a scoring function to select an optimal location from the anonymous collection of location semantics instead of the original location for publishing. Experimental results show that the quality of service of DPALS outperforms the current optimal DP3-SLOC by 7.8%.
(2) A Personalized Differential Privacy for Semantic Trajectory (PDPST) is proposed for the semantic inference attack when trajectories are a continuously queried service. The method first constructs an anonymous set of trajectories according to the privacy protection requirements set by users; it then finds a trajectory with the highest similarity by constructing a trajectory type vector using cosine similarity in the anonymous set. Finally, we introduce an adjustable Gaussian mechanism to add noise to the frequency of semantic type visits in the optimal trajectory according to the user’s personalized semantic type privacy budget. Experimental evaluation shows that the quality of service of PDPST outperforms the current optimal LSBASC by 11.2%.

The rest of this paper is arranged as follows. In Section 2, we introduce the mechanisms associated with our work. Then, notations and preliminaries adopted in this work are described in Section 3. In response to the possible semantic attacks on the single-point location query service, Section 4 proposes a differential privacy protection method for location semantics. To address the possible semantic inference attacks in the trajectory continuous query service, Section 5 presents a personalized differential privacy protection method for semantic trajectories. The conclusions and future work are presented in Section 6.

## 2. Related Work

Numerous researchers have proposed many solutions to the location privacy leakage problems mentioned above. There are three main types of solutions: spatial anonymity-based, encryption-based, and location-distortion-based location privacy protection solutions.

### 2.1. Location Privacy-Preserving Methods

**Spatial anonymity** mainly hides the user’s location, sets the corresponding level of anonymity parameters, and obfuscates the user’s original and anonymous values to protect the user’s location privacy. Commonly used anonymity privacy protection algorithms include *k*-anonymity [4,5,6,7] and rely more on trusted third-party servers to extend the user location sent to the LBS server to include the user’s actual location and k-1 other obfuscated locations to achieve the effect of providing user location privacy. However, the anonymity parameters of this method are difficult to set, and the data availability after anonymization could be better.

**Encryption approaches** usually occur before the user sends location data to the LBS provider and before the LBS provider returns the results to the user, using relevant cryptographic encryption techniques to avoid the user disclosing location privacy during the use of the corresponding search service. The two most common encryption algorithms are based on spatial transformation techniques [8] and privacy information protocols [9]. These cryptography-based privacy protection algorithms can provide relatively strong privacy protection. However, the disadvantage is that they have high storage and arithmetic power consumption, which significantly affects the performance of the terminal.

**Distortion-based** location privacy protection methods are usually performed by pseudonymizing, randomizing, and fuzzifying the location information uploaded by users during their searches using LBS. Dini et al. [10] proposed the generation of fake location data in a specific region randomly. Then, Huang et al. [11] improved on the above algorithm by proposing an algorithm for generating smart fake locations; this algorithm first generates fake locations in place of real locations, and then generates new locations in place of the real locations using Gaussian distribution, thus making it impossible for the attacker to infer the real location of the user. Although the above algorithm is able to protect the user’s location information, it still has some drawbacks. It does not allow a strict definition and adjustment of the privacy budget in the use process. The introduction of the differential privacy (DP) [12,13,14,15,16,17,18,19] protection mechanism, which is a method proposed by Dwork [20] in 2006 based on the security problem of statistical databases, is a good solution to this problem. The advantage of the DP is that it can prove its security with a strict privacy budget; in theory, even if the attacker has some background knowledge, he cannot infer the true information about the user. The core of the algorithm is that it adds noise to the user’s real location. However, the algorithm’s shortcomings are that it needs to easily balance privacy protection and service availability and it only perturbs the location without considering the semantic information.

**Differential privacy** has recently been widely used in privacy protection for location perturbation. Because this protection mechanism can resist background knowledge attacks [20], even when an attacker obtains semantic background knowledge related to the user’s location, he can only infer the user’s location information with a certain probability. Ashwin et al. [21] proposed a fake data generation algorithm to publish the check-in location information for a commute instead of the real location while satisfying different privacy requirements. Ho et al. [22] used a quadtree spatial decomposition technique to ensure DP in databases for location pattern mining. The DP protection mechanism can successfully be applied to location privacy protection in the case of the aggregation of multiple publication users. However, DP requires that any location change has a negligible impact on the magazine. Thus, it does not convey any useful information to the service provider. To overcome this problem, Dewri et al. [23] proposed a new privacy protection scheme by combining the DP protection mechanism with a location anonymization mechanism, which requires a fixed *k*-anonymity set and requires that the probability of reporting the same fuzzy location from any of these *k* locations should not exceed a threshold value $e^{\varepsilon }$. However, there are some problems with this algorithm. First, the privacy-preserving results are related to the selection of the anonymity set. Second, because the published locations are the geometric median of *k* locations, the privacy guarantee is significantly lower than that of the Laplace mechanism.

Andres et al. [16] used the Planar Laplace (PL) mechanism on top of the Laplace mechanism for DP. They proposed geo-indistinguishability, which made DP mechanisms a milestone in location privacy protection. Chatzikokolakis et al. [24] proposed a general approach for conforming to geo-indistinguishability to provide the best quality service in any environment within a reasonable privacy budget. Research was conducted on the problem by analyzing the impact of frequency updates on the privacy level of four mechanisms to address this issue, resulting in an improvement in the standard mechanism for continuous location updates, a common planar Laplace mechanism applicable to sparse locations, and three adaptive mechanisms. Dhubhani et al. [25] proposed an adaptive location protection mechanism that uses the correlation between the user’s location and the previous fuzzy location to add the amount of noise to personalize the user’s location. Zhao et al. [26] summarized specific privacy models and mechanisms together with possible challenges. They also discussed their privacy guarantees against AI attacks and utility losses. To address location data, Zhao et al. [27] proposed a geo-ellipse-indistinguishability privacy notion. As an instantiation of metric differential privacy, geo-ellipse-indistinguishability guarantees pairwise inputs cannot be distinguishable with the level proportional to the privacy budget and Mahalanobis distance between them, given a randomized output. They also presented elliptical privacy mechanisms based on gamma and multivariate normal distributions to achieve this privacy definition. The literature [28] proposes a user-centric location privacy protection mechanism that specifies clusters that satisfy geographic indistinguishability, which creates obfuscated clustering and reduces nearby locations to a single point location. This approach protects the user from single reported location points and continuous reports over time. Today’s location privacy protection algorithms rarely consider the correlation between the user’s location and the duration of movement, which is vulnerable to inference attacks. Based on this, Xiao et al. [29] proposed a solution for the case of high privacy protection degree requirements, where it was shown that there are certain bounds on the errors in time and location in the protection mechanism of differential privacy. The reference [30] filters the trajectory data and filters out the added noise based on the characteristics of Laplacian noise to obtain a dataset similar to the real dataset, which improves the probability that the user’s location privacy will suffer from leakage. The literature [31] uses the self-time autocorrelation function for the above filtering attack to generate a noisy sequence identical to the real trajectory and superimposes it on the real trajectory. The literature [32] proposes a personalized spatio-temporal data privacy protection model based on spatio-temporal data privacy protection (p,q,ε) anonymity, where users can personalize privacy protection parameters according to their preferences. The literature [33] addresses the uncontrollable noise generated by the Laplacian mechanism, demonstrates that the restriction mechanism usually does not preserve the generated noise points when using parameters of a pure Laplacian mechanism, and also proposes a robust method to compute the optimal parameters for satisfying DP under such boundary restrictions.

### 2.2. Summary

Because of the desire to hide the accurate position of an individual’s location, existing methods cannot preserve a sufficient privacy degree and introduce a low-level utility. To remedy this problem, we attempt to propose a practical mechanism to release location semantics while realizing personalized privacy preservation. Specifically, we attempt to address the following challenges:

- Perturbing the location semantics in text form based on DP while preserving the position privacy;
- Calculating the sensitivities of different location semantics and realizing individual’s personalized location semantics protection;
- Designing the mechanism to satisfy the need for personalized location semantics protection.

## 3. Preliminaries

This section first introduces the definition of DP, the nature of the combination, and the noise mechanism of DP. It paves the way for the privacy protection algorithms proposed in this paper.

### 3.1. Differential Privacy

DP was originally a concept applied to statistical databases to protect personal data while publishing aggregated information from the database. The basic idea is that modifying a piece of data in the original database has a negligible impact on the output. DP has a strict definition in theory and is mainly used in data mining, network security, federal learning, statistics, etc. It has become one of the more general privacy protection methods for privacy protection and has a good effect for protecting location data.

#### 3.1.1. Definition of DP

**Definition** **1 (ε-DP)** [20]**.** *Given two datasets* D*,* $D^{'}$ *differing by one record, and supposing* S *is any subset of* R *, let* M *be a random function, and* S *is the set consisting of all possible output values if the random function* M *satisfies Equation (1).*

(1) $$P[M(D)\in S]\leq e^{\varepsilon }P[M(D^{{}^{'}})\in S]$$

**Figure 1:**
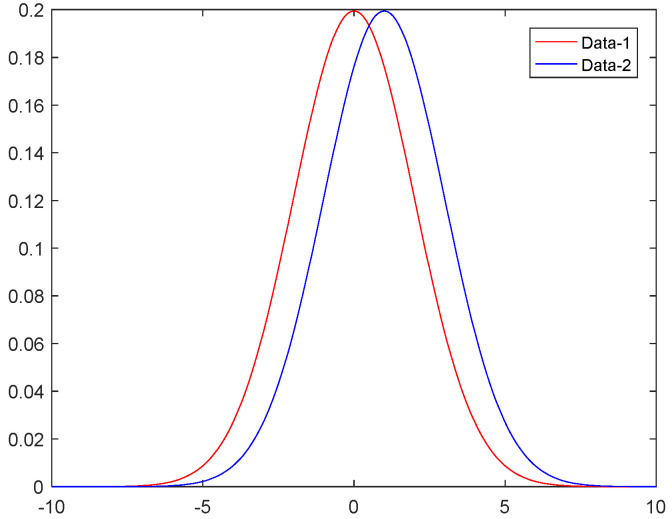
Output probabilities of randomized algorithms on adjacent datasets.

Then, the function M is said to satisfy ε-DP, the parameter ε denotes the privacy protection budget, and P[⋅] denotes the probability of the function M for two datasets D and $D^{\prime }$. DP has a strict mathematical theory defined to ensure that the probability distribution of the output is negligible for two adjacent datasets, regardless of whether they contain a particular record or not, and the privacy budget ε determines the error of the output probability distribution. Figure 1 illustrates the most basic DP model.

The size of the privacy budget ε is an important indicator of the degree of privacy protection. The smaller the size, the smaller the error in the distribution of the output probabilities of two adjacent datasets, the more difficult it is for an attacker to obtain the true location, and the more effective the privacy protection.

Table 1 represents a medical dataset D [34] indicating whether the user has cancer. The diagnosis result is 1 if the user has cancer and 0 if the user does not. If this dataset is externally accessible, the specific diagnosis data information is not visible. We set the query function f(n,i), where n denotes the first n rows of the diagnosis result, and i indicates whether the user has cancer or not. Now we execute a query to find out how many of the first five rows of diagnoses have cancer, f(5,1)=3. If we want to infer whether Alice has cancer, we can directly query the number of diagnoses with cancer in the first four rows of diagnoses by f(4,1)=2.

If f is a query function satisfying DP, $f^{\prime }(n,i)=f(n,i)+noise$, where noise denotes a random noise satisfying some probability distribution. Assume that the output of (2, 2, 3, 4) is f(5,1), then the output of (2, 2, 3, 4) with almost the same probability is f(4,1), so no inference can be made about whether Alice has cancer from the difference between the two outputs.

#### 3.1.2. Noise Mechanisms for DP

There are different noise addition mechanisms for different types of data queries. If the data that noise is to be added to is numerical, such as geographic location data, the Laplace mechanism or Gaussian mechanism is used; if it is non-numerical, the exponential mechanism is generally used. The following is an introduction to each of the noise mechanisms.

**Definition** **2 (Global Sensitivity).** *Given two adjacent datasets* D *and* $D^{\prime }$ *, with a query function* f(⋅) *, the global sensitivity can be expressed as* Δf *, and the value of* Δf *is as follows.*

(2) $$\Delta f=\underset{D,D^{\prime }}{\max}{\left\|f(D)-f(D^{\prime })\right\|}_{1}$$

*where* $\left\|f(D)-f(D^{'})\right\|$ *denotes the Harmattan distance of* f(D) *and* $f(D^{\prime })$*. The global sensitivity is the key parameter for adding the noise size.*

1. Gaussian mechanism

Compared with the Laplace mechanism, which strictly satisfies DP, the Gaussian mechanism provides a relaxed DP mechanism that allows DP to be satisfied within a certain error range class.

**Definition** **3 ((ε,δ)-DP).** *For any* δ∈(0,1) *,* $\delta >\frac{\sqrt{2ln(1.25/\delta )}\Delta f}{\varepsilon }$ *, random function* M*, adjacent datasets* D *and* $D^{'}$ *, if there is noise* $Y\sim N(0,\sigma^{2})$ *satisfying* (ε,δ)*-DP, then:*

(3) $$P[M(D)\in S]\leq e^{\varepsilon }P[M(D^{'})\in S]+\delta$$

*where* δ *denotes the relaxation term, e.g., set to* $10^{-5}$*, indicating that there is at most* $10^{-5}$ *that DP is not respected.* σ *denotes the standard deviation of the Gaussian distribution, which determines the scale of the generated noise. The global sensitivity* Δf*,* $\Delta f=\underset{D,D^{\prime }}{\max}{\left\|f(D)-f(D^{'})\right\|}_{2}$ *, denotes the Euclidean distance between the neighboring datasets.*

**Definition** **4 (Gaussian mechanism).** *For the query function* f(D) *, the result returned is Equation (4).*

(4) M(D)=f(D)+Y

2. Exponential mechanism

In real life, many query operations return non-numerical data, e.g., the query outputs the elements of a set of discrete data $\left\{R_{1},R_{2},\ldots ,R_{n}\right\}$. Based on this, McSherry et al. [35] proposed the index mechanism: when a query is received, instead of a result being output deterministically, the result is returned with a certain probability, thus achieving DP. Moreover, the probability of this output depends on the scoring function, and the higher the score, the higher the probability of the output.

**Definition** **5 (Exponential mechanism).** *Suppose there is a dataset* D *and set object* R*; let the output object of a random function* M *be* $R_{i}\in R$ *, and the scoring function be* $q(D,R_{i})$*. If the random function* M *outputs the result with probability* $M(D,q,R_{i})\sim e^{\frac{\varepsilon q(D,R_{i})}{2\Delta q}}$*, it is said that the random function* M *satisfies the exponential mechanism of DP, where* Δq *denotes the global sensitivity, as in Equation (5).*

(5) $$\Delta q=\underset{D,D^{'}}{\max}{\left\|q(D,R_{i})-q(D^{'},R_{i})\right\|}_{1}$$

It can be concluded from the above analysis that, when the privacy budget ε is large, objects with higher scoring functions are more likely to be output. When the privacy budget ε is small, the difference in the probability of the outputting scoring functions for each object becomes smaller. It tends to disappear as the privacy budget decreases.

#### 3.1.3. Combined Characteristics of DP

**Property** **1 (Post-processing property [36]).** *if an algorithm* $M_{1}(\cdot )$ *satisfies* ε*-DP, then for any algorithm* $M_{2}(\cdot )$*, the combined algorithm* $M_{1}(M_{2}(\cdot ))$ *also satisfies* ε*-DP.*

**Property** **2 (Serial composition1 [37]).** *as in* Figure 1 *let randomized algorithms* $M_{1},M_{2},\ldots ,M_{n}$ *all satisfy DP; their privacy budgets* $\varepsilon_{1},\varepsilon_{2},\ldots ,\varepsilon_{n}$ *are then for the same dataset* D*. The combination of these algorithms* $M(M_{1}(D),M_{2}(D),\ldots ,M_{n}(D))$ *provides* $\sum_{i=1}^{n}\varepsilon_{i}$*-DP.*

Serial combinatoriality illustrates that the level of privacy protection of a serial algorithm consisting of multiple algorithms that conform to DP is the sum of the privacy protection budgets of all algorithms. Serial combinatoriality applies to the same dataset and query operations consisting of different lookup void functions.

**Property** **3 (Parallel composition [38]).** *as in* Figure 1 *assume that there are algorithms* $M_{1},M_{2},\ldots ,M_{n}$ *that satisfy DP, that the individual privacy budgets of the algorithms are* $\varepsilon_{1},\varepsilon_{2},\ldots ,\varepsilon_{n}$*, respectively, for n datasets without intersection* $D_{1},D_{2},\ldots ,D_{n}$*, and that the combination of these algorithms* $M(M_{1}(D_{1}),M_{2}(D_{2}),\ldots ,M_{n}(D_{n}))$ *provides* $\underset{1<i<n}{\max}\varepsilon_{i}$*-DP protection.*

## 4. DP Preserving for Single Point Location Semantics

In this section, we propose a DP protection algorithm for location semantics, which is not considered in existing location privacy protection algorithms. The algorithm is based on the “geographic indistinguishability” framework. It sets the semantic privacy level protection parameters, adds the generated noise to the real location, and constructs the LSS using the optimal location semantics based on the exponential mechanism. Furthermore, the exponential mechanism calculates the semantic area with the highest output probability in the LSS as the published location. The effectiveness of the proposed algorithm in terms of privacy protection degree, quality of service, and computational overhead is verified by experiments.

### 4.1. System Architecture

**Figure 2:**
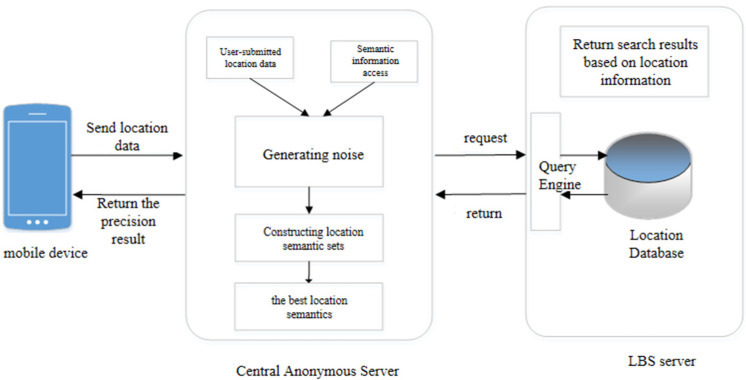
The system architecture of the DP protection method with location semantics.

Considering the performance of user mobile devices and the lack of storage space, this paper adopts a centralized location privacy protection architecture, as shown in Figure 2. This location privacy protection architecture is divided into three parts, the mobile device, the central anonymous server, and the LBS server. The premise of this architecture is to have a trusted central anonymous server. When a user initiates a query request, they first obtain the location information about the mobile device through positioning technology and send the real location information and the query request information to the central anonymous server. The central anonymous server receives the location information and requests information from the mobile device, queries the semantic information stored on the server, and determines the semantic location range. Finally, the user sets the corresponding semantic privacy level protection parameters and the corresponding noise, adds the generated noise to the real location sent by the mobile device, builds an LSS to meet the privacy requirements, and selects an optimal location semantics and requests information to send to the LBS server. The location server returns a candidate set of query results based on the sent query request and alternative location information. The central anonymous server runs an improvement filter over the candidate set and returns the results to the mobile device.

### 4.2. Location Semantic Attack Model and Problem Definition

#### 4.2.1. Location Semantic Attack Model

**Figure 3:**
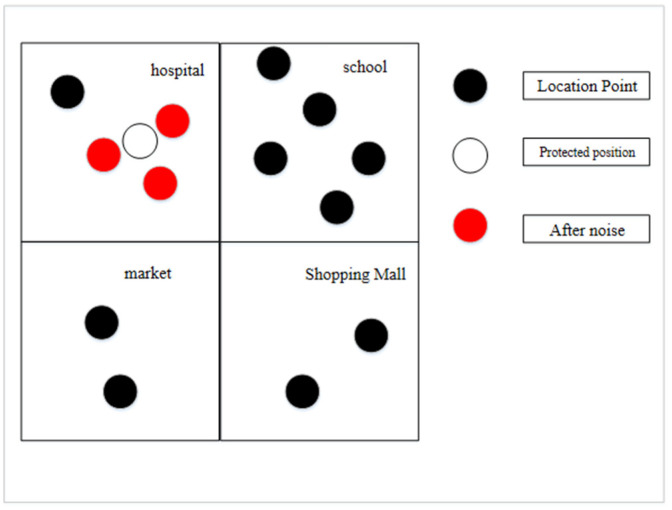
Location semantic attack model.

The location semantic attack model refers to the fact that when locations are anonymized, if all the locations in the anonymization set are of the same semantic type, the attacker will infer the user’s location privacy information based on the semantic type if he has background knowledge related to the semantic type. As shown in Figure 3, when the original location uses the privacy protection mechanism of adding noise, the perturbed locations may become the same semantic type after the noise is added. Suppose these perturbed locations are used instead of the real locations. In that case, the attacker will infer private information about the user’s health status from the semantic information, such as the semantic information about the hospital.

#### 4.2.2. Problem Definition

**Definition** **6 (Physical Location (Location)).** *The physical location usually refers to the location of the user in terms of longitude and latitude, and* L(x,y) *represents the physical location of the user, where the longitude and latitude of the user are represented by* x *and* y*, respectively.*

**Definition** **7 (Location Semantic (LS)).** *Location usually includes physical location and location semantics, and physical location refers to the longitude and latitude at a certain coordinate. Location semantics refers to the location with features such as longitude, latitude, and semantic types, such as supermarket, government, school, and other gathering areas. This study uses*  SL(id,lat,lng,semid,fr) *to denote the location semantics of the user, where* id *is used to identify the user;* lat *and* lng *denote the longitude and latitude of the user’s location, respectively;* semid *denotes the semantic type information of the user’s location;* fr *denotes the total number of times the semantic type of the location has been accessed.*

**Definition** **8 (Location Semantic Set (LSS)).** *The LSS is obtained using the optimal location semantic selection algorithm based on the exponential mechanism according to the user’s location. Only some of the semantic types in this LSS are the same. The set of semantic locations is denoted by*LSS*, and the set of location semantics obtained by the final algorithmic solution of a location is denoted by*  $LSS=(LS_{1},LS_{2},\ldots ,LS_{n})$.

**Definition** **9 (Semantic Sensitivity).** *Semantic sensitivity refers to the sensitivity of the semantic type in the location semantics. The semantic type in the location semantics is proportional to the semantic sensitivity, which generally takes a value between 0 and 1. We denote the semantic sensitivity as*SS*, and we denote the set of sensitivities of different types of positional semantics in n as*$SS=(sen_{1},sen_{2},\ldots ,sen_{n})$.

**Definition** **10 (Physical Location Distance (Location Distance)).** *Location is generally divided into geographic location and location semantics. This algorithm for location distance is mainly used to calculate the physical distance and the distance between the physical location in two semantic locations. It uses the Euclidean distance to calculate the straight-line distance between the two location semantic centers, as shown in Equation (6).*

(6) $$D_{euc}(L_{i},L_{j})=\sqrt{{\left(x_{j}-x_{i}\right)}^{2}+{\left(y_{j}-y_{i}\right)}^{2}}$$

*where* $\left(x_{i},y_{i}\right)$ *and* $\left(x_{j},y_{j}\right)$ *denote the coordinates of semantic location points* $L_{i}$ *and* $L_{j}$.

**Definition** **11 (Privacy Requirement).** *Privacy Requirement is denoted by* PR *in this paper and* PR={STN,SSN} *, where* STN *denotes the privacy semantic type requirement metric, and* SSN *denotes the semantic sensitivity requirement metric.*

**Definition** **12 (Semantic Type Set (STS)).** *The semantic type set is a semantic feature in location semantics, which consists of different semantic types. This definition is mainly applied to the user’s privacy requirements, and in the algorithm the user’s privacy budget is mainly the semantic type set, where* $STS=(S_{T}{}_{1},S_{T}{}_{2},\ldots ,S_{Tn})$ *is used to denote* n *semantic location type sets.*

**Definition** **13 (Geo-Indistinguishability [16]).** *Geo-indistinguishability takes the real location as a circle with a radius of* r*. All users within this circular region can enjoy* εr *-privacy protection, which is easiest for the user to demand as a pair* (l,r) *, with* l *denoting the privacy budget,* l=εr.

The researchers transformed the one-dimensional data privacy protection into two-dimensional data privacy protection using the processes of coordinate transformation, data discretization, and mapping to form a planar Laplace mechanism. The noise mechanism based on geographic indistinguishability is:

(7) $$D_{\varepsilon }(r,\theta )=D_{\varepsilon ,R}(r)\cdot D_{\varepsilon ,\Theta }(\theta )$$

where $D_{\varepsilon ,R}$ and $D_{\varepsilon ,\Theta }(\theta )$ are independent of each other and are calculated as shown in Equations (8) and (9), respectively.

(8) $$D_{\varepsilon ,R}(r)=\int_{0}^{2\pi }D_{\varepsilon }(r,\theta )d_{\theta }=\varepsilon^{2}re^{-\varepsilon r}$$

(9) $$D_{\varepsilon ,\theta }(\theta )=\int_{0}^{\infty }D_{\varepsilon }(r,\theta )d_{r}=\frac{1}{2\pi }$$

It can be seen that $D_{\varepsilon ,R}(r)$ corresponds to the probability density function of the gamma distribution with shape 2 and scale $\frac{1}{\varepsilon }$. Because R and Θ are independent, the most efficient way to find the tuple parameters (r,θ) in function $D_{\varepsilon }(r,\theta )$ is to calculate $D_{\varepsilon ,R}(r)$ and $D_{\varepsilon ,\Theta }(\theta )$ independently.

As $D_{\varepsilon ,\Theta }(\theta )$ is a constant, the most efficient method is to generate a uniformly distributed random number θ in the interval [0,2π]. For $D_{\varepsilon ,R}(r)$, consider its cumulative distribution function as follows:

(10) $$C_{\varepsilon }(r)=\int_{0}^{r}\varepsilon^{2}\rho e^{-\varepsilon \rho }d\rho =1-(1+\varepsilon r)e^{-\varepsilon r}$$

$C_{\varepsilon }(r)$ denotes the probability of a random point falling in the radius interval [0,r]. A uniformly distributed random number z is first generated in interval [0,1), and then we set $r=C_{\varepsilon }^{-1}(z)$ to transform it to obtain the equation shown in Equation (11) where $W_{-1}$ denotes the lambertW function-1 branch.

(11) $$r=C_{\varepsilon }^{-1}(z)=-\frac{1}{\varepsilon }(W_{-1}(\frac{p-1}{e})+1)$$

Given a Cartesian coordinate system and an actual physical location L=(x,y), it is only necessary to independently generate the noise tuple. The location point after adding the noise is $L^{\prime }=(L+r\cos\theta ,L+r\sin\theta )$.

### 4.3. DP Preservation Methods for Location Semantics

#### 4.3.1. Algorithm Description

Conventional distortion-based location privacy protection algorithms do not consider the semantic information of location, thus making it easy for attackers to obtain users’ location privacy information based on semantic inference attacks. For example, when noise is added to the user’s location with the common location perturbation methods, only a small range of data is perturbed. However, the semantic type of the location data and the real location data after perturbation are still the same, so the security of location information privacy cannot be guaranteed. The Differential Privacy protection Approach for Location Semantics (DPALS) algorithm proposed in this paper considers the semantic type information while perturbing the location. It can defend well against semantic inference attacks by attackers.

To ensure security when constructing the semantic type set, this algorithm uses the index mechanism based on “geographic indistinguishability” and DP in location generation and selection. “Geographic indistinguishability” is mainly used to perturb the location and generate new location semantics, and the exponential mechanism is used to select the best location semantics in the set of location semantics. The main steps of the algorithm are as follows.

(1) The semantic type of the user’s location and the number of times the semantic type has been accessed can be obtained from the user’s location semantics, and the location semantics is added to the LSS.
(2) The set of location semantics is constructed by the optimal location semantics selection algorithm based on the exponential mechanism and using the “geographic indistinguishability” mechanism to generate noise for the user’s location semantics. The semantic type in the location semantics is the semantic type that corresponds to the location after perturbation.
(3) Determine whether the location semantics in LSS meets the privacy requirement PR, and if it does, go to the next step; if not, repeat steps (2) to (3).
(4) The optimal location semantics is selected from the location set according to the optimal location semantics selection algorithm based on the exponential mechanism.

Indeed, in our solution, we first select nearby location semantics around the individual’s real location to build the candidate semantics set. Then we select the optimal location semantics in the set to publish based on the exponential mechanism. If there is no location semantics around the user, we will expand the search range around the individual’s location until we find a specific location semantics. Thus, the result set is always not null. In addition, in theory, a closer semantics has a good data utility, and bad privacy protection degree, and vice versa.

The DPALS algorithm first uses the noise mechanism of “geographic indistinguishability” to obtain the location semantics. Then, the semantic types and access frequencies of the locations are selected, forming multiple location semantics, including the real location. This is done to expand the location point selection area, which is not limited to a small range, and to help improve the success rate of semantic recognition. The pseudo-code of the DPALS algorithm is shown in Algorithm 1.
**Algorithm 1.** DPALS algorithm pseudo-code.**Input:user Location Semantics** 
LS(id,lat,lng,semid,fr)
**, Privacy Requirement** 
PR

**Output:** 
LSS
1. Initialize variables: semantic location set LSS={∅}, Semantic Type Set STS={∅};2. Determine the semantic location of the user based on the location of the user: $LS_{u}$, semantic type $ST_{u}$;3. $LSS=\{LS_{u}\}$, $STS=\{ST_{u}\}$; //User semantic location and location type are added to the corresponding sets4. *n* = 1;//Set a count variable that marks the semantic type5. **while** (*n* < STN)6.  draw θ unif. in [0,2π);//Generate clip angle7.  draw p unif. in [0,1);8.  $r=C_{\varepsilon^{'}}^{-1}(p)$;//Generate radius9.  $L^{'}{}_{u}=L_{u}+(r\cos(\theta ),r\sin(\theta ))$;//Add noise to the physical location, i.e., latitude and longitude in $LS_{u}$10.  $LS_{u^{'}}=(\cdot ,L_{u^{'}}(x),L_{u^{'}}(y),semid^{'},fr^{'})$;//Generate the semantic position after adding noise11.  For the number of visits $fr^{'}$ in $LS_{u^{'}}$, combined with the privacy requirements PR(SSN);12.  Calculate $q(LS_{i})$;13.   Calculate the probability $p({LS}_{i})$; 14.   **if** $fr^{'}>SSN$
**and** STS **does not contain** $semid^{'}$//If greater than the semantic sensitivity requirement and the semantic type set does not contain the current semantic type15.    $LSS=LSS\cup LS_{u^{'}}$, $STS=STS\cup semid^{'}$;//Add semantic location and semantic type to LSS and STS respectively 16.    *n*++;//Number of semantic types plus one17.   **end if**18. **end while**19. Obtain the optimal set of semantic locations LSS;20. Select the semantic position having the highest probability from LSS according to the optimal semantic position algorithm based on the exponential mechanism;21. **return**
$LS_{p_{\max}}$

In this pseudo-code, step 1 initializes the set of location semantics and semantic types, step 2 adds the user’s location semantics and semantic types to the set of location semantics and semantic types, and steps 3 to 19 construct the set of location semantics that meets the privacy requirements. Steps 20 to 21 compute and obtain the best location semantics.

#### 4.3.2. Design of Scoring Function for Exponential Mechanism

This study uses the exponential mechanism to select the optimal location semantics from the set of constructed semantic locations. Because the exponential mechanism is consistent with the idea of DP, it is more secure and less susceptible to background knowledge attacks when used. It can also ensure the privacy requirements are met according to the scoring mechanism when it selects the best location semantics.

When the exponential mechanism is used to select the best positional semantics, how the scoring function is set is the key to the final selection result. In this section, the scoring function is represented by $q(LSS,LS_{i})$, where LSS denotes the set of positional semantics constructed by the algorithm based on the exponential optimal positional semantics selection algorithm, and $q(LSS,LS_{i})$ is the score of the i th positional semantics of the positional semantics set LSS, which is calculated as shown in Equation (12).

(12) $$q(LS_{i})=\frac{SS_{SL_{i}}*fr_{SL_{i}}+D_{euc}(L,L_{i})}{2}$$

In Equation (12), the concept of semantic sensitivity is introduced and combined with the physical location distance between location semantics and the user-initiated location semantics to calculate the score of semantic location.

#### 4.3.3. Optimal Location Semantic Selection Algorithm

To illustrate how optimal location semantics is selected from the set of location semantics, this section proposes an optimal location semantics selection algorithm based on an exponential mechanism. The pseudo-code of this algorithm is shown in Algorithm 2, and the main steps of the algorithm are as follows:

(1) According to Equation (7), the score of each positional semantics set in the positional semantic set LSS can be obtained. Then the weight $W(LS_{i})$ of each positional semantics is calculated as follows:

(13) $$W(LS_{i})=e^{\frac{\varepsilon *q(LS,LS_{i})}{2*\Delta q}}$$

where ε denotes the privacy budget given when semantics are selected for the LSS, and Δq denotes the difference between the real user location $LS_{u}$ and the current location semantics $LS_{i}$, as shown in Equation (14).

(14) $$\Delta q=\max{\left\|q(LS_{i})-q(LS_{u})\right\|}_{1}$$

(2) Based on the weights derived from Equation (16), the probability of selecting each location semantics is calculated using the DP index mechanism. The calculation results are ranked from largest to smallest, and the calculation is shown in Equation (15).

(15) $$p(LS_{i})=\frac{W(LS_{i})}{\sum_{LSj\in LSS}W(LS_{j})}$$

(3) The position semantics having the highest probability is selected as the optimal position semantics $LS_{u^{'}}$.

**Algorithm 2.** Pseudo-code of the optimal semantic location selection algorithm.**Input:Semantic Location** 
LSS
**,** 
PR(SS)
**Output: Optimal Semantic Location** 
$LS_{u^{'}}$
1. Initializing variables: semantic position sets $LSS=\{LS_{u}\}$;2. **for** each $LS_{i}\in LSS$3.  Calculate the score function according to Equation (14): $q(LSS,LS_{i})$;4.  Calculate the weights according to Equation (15): $W(LS_{i})$;5.  Calculate the probability according to Equation (17): $p(LS_{i})$;6.  Compare and update $p(LS_{i})$, and get the semantic position corresponding to the largest p;7. **end for**8. **return**
$LS_{p_{\max}}$

#### 4.3.4. Algorithm Analysis

The DPALS algorithm proposed in this section is based on the mechanism of “geographic indistinguishability” to generate location semantics after adding noise, and it sets privacy requirements when constructing the LSS, combines semantic types and semantic sensitivities, and introduces an exponential mechanism to ensure security. The exponential mechanism is one of the methods for realizing DP. One of the advantages of DP is that it gives the upper bound of information leakage probability. In other words, the user can ignore the attackers’ background knowledge about the location. Even if the attacker has all of the background knowledge of the user, e.g., gender, age, and job occupation, the exponential mechanism can still provide the privacy guarantee that it claims. The privacy degree does not change along with the background knowledge that the attacker has. Due to the setting of semantic types, not only is the location perturbed, it can also resist the semantic inference attack of attackers and reduce the probability of the user’s location semantic information being leaked. Compared with the traditional DP, *k*-anonymity, and *l*-semantic diversity location privacy protection mechanisms, the DPALS algorithm provides more comprehensive protection.

### 4.4. Experiment and Analysis

#### 4.4.1. Experimental Setting

(1) Experimental environment

The experiments were conducted on Windows 10 using PyCharm software and the Python language, with the following hardware environment: CPU Intel i5 4500u, 16 GB RAM.

(2) Experimental data

**Figure 4:**
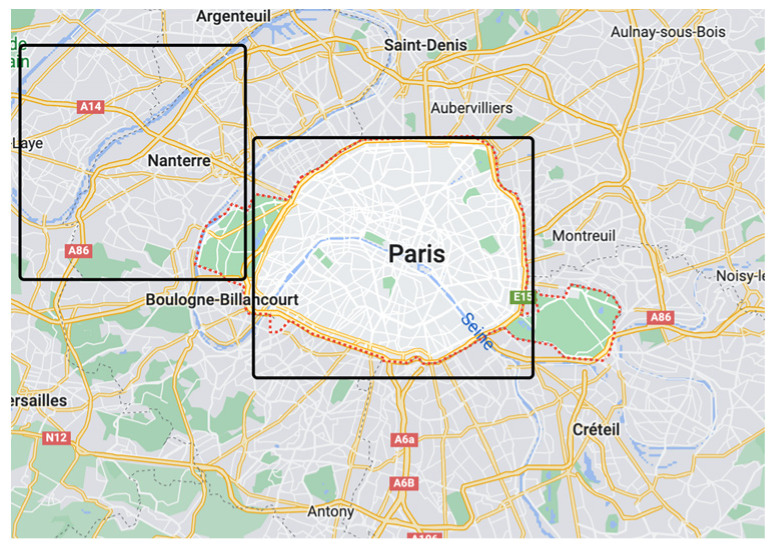
Privacy protection zones: city of Paris and Nanterre district.

Two open datasets were chosen for the experiment, and the selected areas were located in the Paris metropolitan area and Nanterre metropolitan area, France, as shown in Figure 4.

The area of the two selected public datasets is shown in the rectangular box in Figure 4. The rectangular box on the right indicates an area of 75 km× 75 km centered on the city of Paris, and the rectangular box on the left indicates an area of 70 km× 70 km centered on the city of Nanterre, which covers the surrounding metropolitan area.

The Gowalla [39] dataset is a location check-in dataset that contains 644,289 check-ins from February 2009 to October 2010 for 196,591 users, with 9635 check-ins for the Paris metropolitan area and 429 check-ins for the Nanterre metropolitan area. The Brightkite [40] dataset contains 4,491,143 check-ins for 58,228 users, with 4014 check-ins for the Paris metropolitan area and 386 check-ins for the Nanterre metropolitan area. These two datasets have better use value than the trajectory dataset because the semantic information of check-in indicates the location of interest to the user and has good mining value. In contrast, the trajectory dataset only contains information about movement without any information about the actual use of the LBS.

#### 4.4.2. Experimental Indicators

The experiment mainly verifies the location data availability of this algorithm in terms of privacy protection, computational overhead, and quality of service.

(1) Privacy Protection Indicators

As the four privacy-preserving mechanisms have different definitions of privacy, the more widespread Bayesian mechanism privacy metric [40] is used for a uniform comparison, which considers a Bayesian attacker who has prior knowledge π of the user’s possible locations and observes the output of mechanism K. After the attacker obtains the published location Z, he uses strategy h:Z→X to remap z to what he believes to be the user’s likely true location. The attacker’s expected loss in this mechanism is defined as:

(16) $$A_{DV}E_{RROR}(K,\pi ,h,d_{A})=\sum_{x,z}\pi (x)K(x)(z)d_{A}(x,h(z))$$

where $d_{A}$ is a loss indicator used to simulate when an attacker is unable to identify the user’s true location, and the loss function is used in the experiments to remap the location h(z).

For the experimental evaluation, the point of interest (POI) of each dataset at the time of privacy and two commonly used loss functions are used to simulate different adversaries: the first is the binary loss function $d_{bin}$, as in Equation (16), which simulates an attacker interested in the semantic information about the user POI, for which $A_{DV}E_{RROR}(K,\pi ,h,d_{bin})$ represents an attacker’s guess that is close to the POI adversary; the second is the Euclidean loss function $d_{euc}$, which simulates an attacker guessing close to the real POI, for which $A_{DV}E_{RROR}(K,\pi ,h,d_{euc})$ represents the attacker’s error in guessing the distance to the user POI [41].

(17) $$d_{bin}(x,z)=\left\{\begin{array}{cc} 0 & x=z\\ 1 & x\neq z \end{array}\right.$$

(2) Service Quality

Quality of service is an essential measure of the availability of location data, and distance is used in experiments to measure the error between the original location and the reported location. When the difference between the original location and the actual location is large, there is a significant degradation in the quality of service, and the use of Euclidean distance can provide a more suitable method [16]. Such a method allows the addition of specific noise without any effect until a certain critical value is reached, after which there is a significant degradation in the quality of service. In this case, the distance function is calculated using Equation (18) to determine service quality.

(18) $$d_{r}(x,z)=\left\{\begin{array}{c} 0,\\ 1, \end{array}\right.\begin{array}{c} d_{euc}(x,z)\leq r\\ other \end{array}$$

(3) Calculated Overhead

The computational overhead represents the time spent by the entire algorithm to complete the process of protecting location data at one time. For the algorithm proposed in this section, the algorithm protection mechanism is divided into the time spent on location semantic acquisition, semantic sensitivity calculation, noise calculation, noise addition, and the combination of sending the location and requesting the corresponding service. The computation overhead is the most intuitive factor in measuring the quality of the service, and the shorter the time spent on the computation overhead, the better, under the premise of ensuring the user’s privacy.

#### 4.4.3. Experimental Analysis

In this section, multi-dimensional experimental simulations are performed on two real public datasets, and the simulated experiments are compared and analyzed with existing mechanisms presented in the literature, namely, PL privacy-preserving mechanisms [28], EM privacy-preserving mechanisms [42], and DP3-SLOC privacy-preserving mechanisms [43] in terms of privacy protection, computational overhead, and quality of service.

(1) Bayesian Attack Query Error

**Figure 5:**
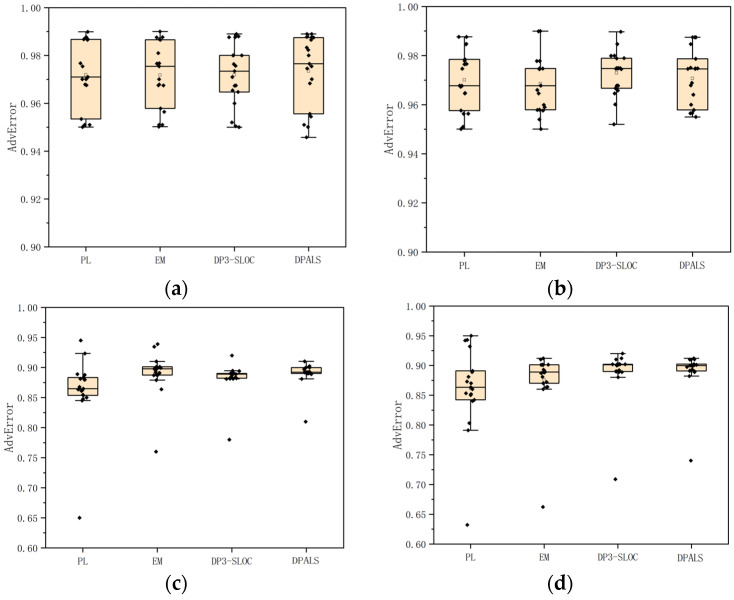
Query error rate of interest points for each region on the two datasets. (**a**) Gowalla Dataset (Paris); (**b**) Brightkite Dataset (Paris); (**c**) Gowalla Dataset (Nanterre); (**d**) Brightcity Dataset (Nanterre).

This experiment simulates two public datasets according to the privacy-preserving metrics proposed in Section 3.1.2. Figure 5 shows the experimental results for the query errors of four privacy-preserving mechanisms on two public datasets according to Bayesian attacks in different regions.

As can be seen in Figure 5a, the level of privacy protection is high for the four privacy protection mechanisms, with an average query error rate of over 94%. This is in line with the reality that the query error is higher because the data were captured in and around the urban area of Paris, where the points of interest are dense. The DPALS algorithm has a better level of privacy protection in this area, and it can be seen that the median and average query error rates are the highest, at 97.56% and 97.03%, respectively. From Figure 5b, it can be seen that the overall privacy protection level of the four mechanisms is high, and the average query error rate is higher than 96%, but the PL privacy protection mechanism fluctuates the most compared with the other three privacy protection mechanisms. This is because the PL mechanism does not have a fine-grained division for the noise when it adds Laplace noise, it does not consider semantic information, and the added noise is random. The overall stability could be better.

Figure 5c,d compares the Bayesian attack query errors using the four privacy-preserving mechanisms in the Nanterre region of the two datasets. From the figures, it can be seen that the DP3-SLOC privacy protection mechanism, EM privacy protection mechanism, and DPALS privacy protection mechanism have better overall privacy protection, and the average query error rate of the three privacy protection mechanisms reaches 89.6% on the Gowalla dataset and 90.4% on the Brightcity dataset. The performance of the PL privacy protection mechanism on the Nanterre data is worse than that of the Paris metropolitan area, with a low query error rate of 63%. This is due to the fact that the Nanterre area has fewer points of interest than the Paris metropolitan area and the distance between points of interest is larger, resulting in the a priori probabilistic information being better known to the attacker, so it is easier for the attacker to distinguish between such points of interest.

Figure 5c,d shows that, with the use of four privacy-preserving mechanisms in the Nantes minefield, there are several instances where the query error is lower, even up to 63%, because certain location points are outliers. The addition of noise has little effect. In terms of outliers, we can take advantage of the abnormal detection methods (e.g., clustering) to filter outliers. Specifically, we can find outliers by combining an individual’s road network and transportation information. For example, we can calculate the speed of the user based on publishing a trajectory’s timestamps and latitude and longitude data. Then, combined with the use of Google Maps, we can know the possible semantics the user can arrive at. In this way, we can filter the outliers. Compared with the dataset without outliers, the query error is bigger than the one with outliers.

(2) Quality of Service

**Figure 6:**
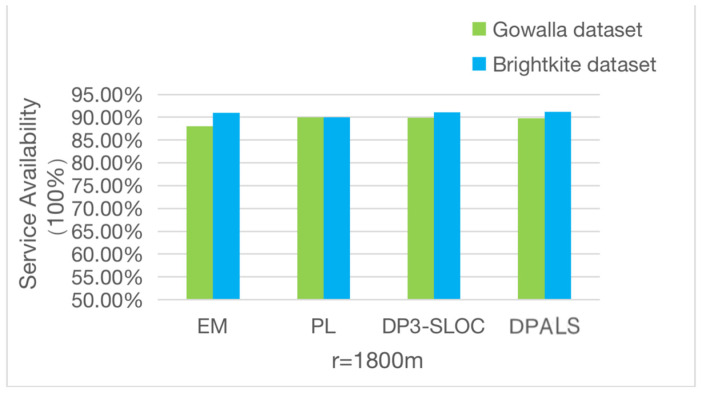
Comparison of quality of service at r = 1800 m.

**Figure 7:**
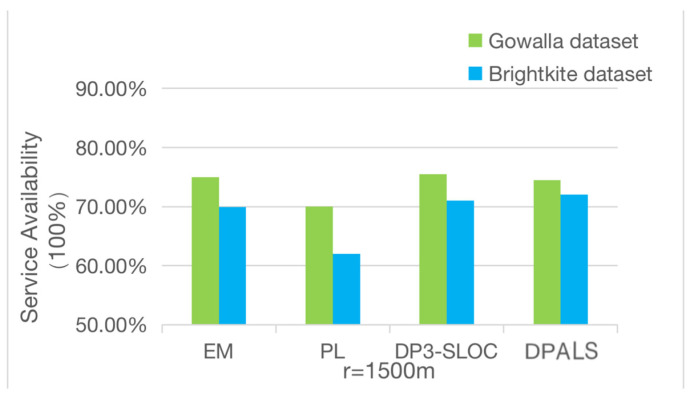
Comparison of service quality at r = 1500 m.

This experiment compares the quality of service of this algorithm based on the quality of service performance metrics proposed in Section 4.3.1. While satisfying the privacy-preserving lower bound, the quality of service changes accordingly with the values r takes. In this experiment, the groupings according to the literature [42] are used, i.e., r= 1800 m, 1500 m, 1200 m, and the results are averaged over 1000 random repetitions. The obtained results are shown in Figure 5, Figure 6 and Figure 7. It can be seen from the figures that the quality of service obtained by the four privacy protection mechanisms differs greatly when different values of r are taken.

As can be seen from Figure 6, when using *r* = 1800 m, the quality of service of the four privacy protection mechanisms is better on both datasets and reaches about 90% on average. These results are in line with the actual situation, and when the distance error is larger than expected, the minimum requirement of privacy protection can be met, and the quality of service can be guaranteed.

As shown in Figure 7, when *r* is 1500 m, the privacy-preserving mechanisms DPALS, DP3-SLOC, and EM achieve a high quality of service on the Gowalla dataset, up to about 75% on average, while PL reaches a level of 70%. However, the quality of service drops to 62% on the Brightkite dataset after the PL privacy protection mechanism. The service quality of the PL privacy protection mechanism inevitably decreases with the decrease in the PL privacy protection mechanism because the DPALS privacy protection mechanism, DP3-SLOC privacy protection mechanism, and EM privacy protection mechanism take semantic information into account when adding Laplace noise. The addition of noise also considers the influence of semantic sensitivity to ensure the service availability. From the experimental results, we can see that when *r* = 1500 m, the service quality of the PALS privacy protection mechanism, EM privacy protection mechanism, and DP3-SLOC privacy protection mechanism are equal, and all three means consider the location semantic information.

**Figure 8:**
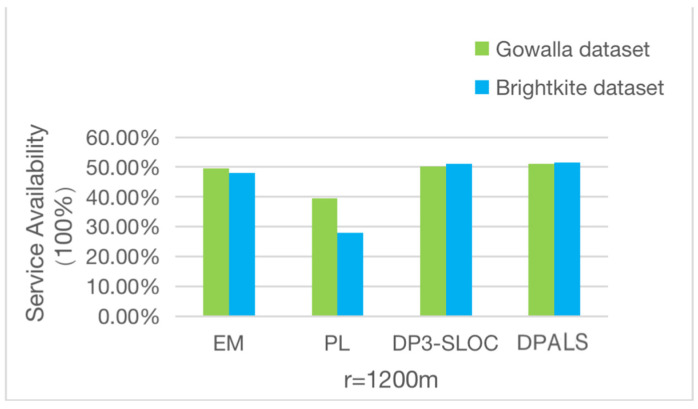
Service quality comparison for r = 1200 m.

The quality of service on the two datasets when *r* is set to 1200 m is shown in Figure 8. There is a significant drop in service availability after processing the PL privacy protection mechanism. The service quality is relatively low and is already below the minimum privacy protection. The data are no longer usable. The other three privacy protection mechanisms have a better quality of service, which reaches 50% on average. This value is in line with the actual situation.

From Figure 6, Figure 7 and Figure 8, we can conclude that when the value of r is less than 1500 m, the PL privacy protection mechanism cannot guarantee the quality of service, and the other three privacy protection mechanisms provide a better quality of service. When the value of *r* is greater than 1500 m, all four privacy protection mechanisms provide a better quality of service, and the DPALS privacy protection mechanism is slightly better than the DP3-SLOC privacy protection mechanism.

(3) Calculated Overhead

**Figure 9:**
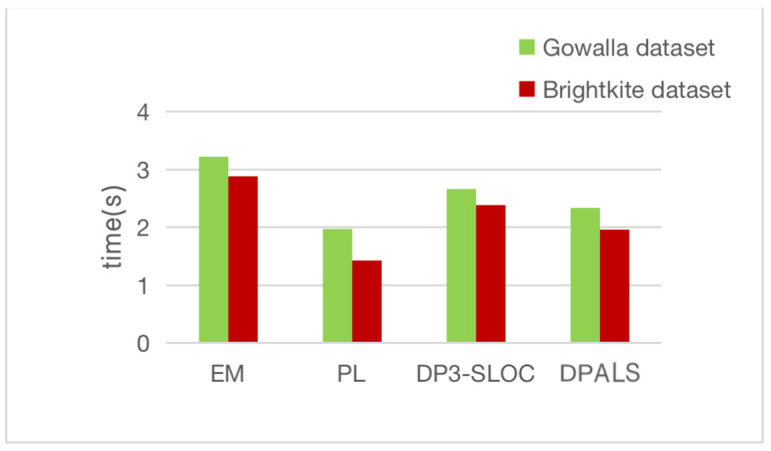
Computational overhead of the four protection mechanisms on the two datasets.

The computational overhead of this experiment is measured as the average of 1000 randomly repeated experiments run on both datasets. Figure 9 shows the time required to execute the EM privacy protection mechanism, the DP3-SLOC privacy protection mechanism, the PL privacy protection mechanism, and the privacy protection mechanism proposed in this section.

As can be seen in Figure 9, the PL privacy protection mechanism takes the shortest time among the four privacy protection mechanisms, with a computation overhead of 1.9908 s on the Gowalla dataset and 1.4402 s on the Brightkite dataset. The PL privacy protection mechanism has the lowest computational overhead, but the quality of service is not guaranteed. The other three privacy protection mechanisms have higher computational overhead, but other metrics are significantly better than PL privacy protection mechanisms.

## 5. Personalized DP Preservation Methods for Semantic Trajectories

When users make continuous queries, they upload their trajectory data at different times, and if attackers obtain the trajectory information at different times, they can infer the user’s location. Considering this, we propose a personalized DP protection method for semantic trajectories by first constructing a trajectory anonymization set according to the user’s privacy requirements. We construct a semantic vector according to the semantic types of the anonymous trajectories, calculate the trajectory similarity using cosine similarity, and obtain a trajectory that is most similar to the original trajectory; finally, we introduce an adjustable Gaussian mechanism to visit each semantic type on the optimal trajectory frequency to add personalized noise to improve the security of the published trajectory. The effectiveness of the algorithm in terms of the degree of privacy protection and data availability is verified through experimental demonstration.

### 5.1. Continuous Query Attack Model

A continuous query attack is a classical query attack. When users make queries at different times, they upload different trajectory information. Because the user’s trajectory is always changing, the generated trajectory anonymous set is also changing. If the attacker obtains several anonymous trajectory sets, the real trajectory of the user can be inferred through the trajectory intersection and the semantic location access of related time points.

**Figure 10:**
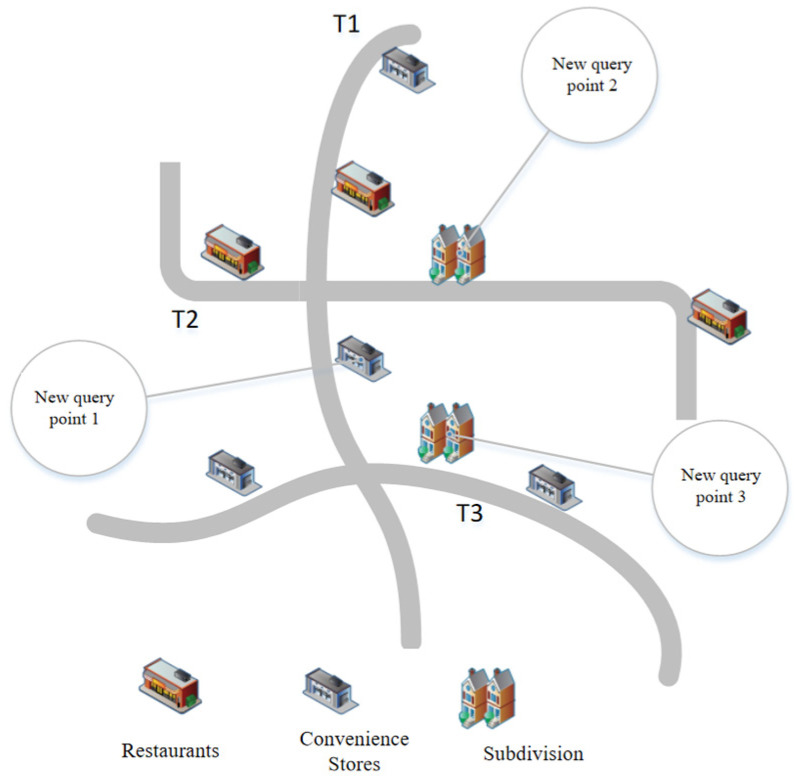
Continuous query attack model.

Users need to upload tracking data when they perform query operations at different times. To obtain the query results in higher quality, it is necessary to upload the semantic types on top of the trajectory and the number of historical visits to the server. Suppose there is no perturbation to the number of visits. In that case, the attacker will infer the location privacy of the user based on the number of semantic types uploaded for the many different queries. Figure 10 shows the continuous query attack model. When the user obtains three convenience store and three semantic restaurant types in the first query operation, a new convenience store and two neighborhood semantic types are added in the second query, and the attacker infers the user’s location as the trajectory T2 by the number of semantic query types and the query time of the neighborhood.

### 5.2. Definition of Problem

**Definition** **14 (Semantic Trajectory (ST)).** *Semantic trajectory refers to the location sequence consisting of location semantics generated by filtering the original trajectory by setting the dwell time threshold, generally by using* $ST=\{LS_{1},LS_{2},\ldots ,LS_{i},\ldots LS_{n}\}$ *to represent it, where* $SL_{i}$ *denotes the* i *th location semantics in the semantic trajectory, which consistent with the location semantics description in Definition 8.*

**Definition** **15 (Trajectory Level Parameter).** *The semantic trajectory level parameter is the number of trajectories that need to be anonymized in the semantic trajectory anonymization set, denoted by*TL.

**Definition** **16 (Trajectory Anonymous Set).** *An anonymous trajectory set is a set of* TL *privacy-compliant trajectories including the user trajectories, denoted by* $TAS=\{ST_{1},ST_{2},\ldots ,ST_{TL}\}$.

**Definition** **17 ( θ-Security).** *Given a trajectory, if the trajectory sensitivity rate* TSR<θ*, then the trajectory meets trajectory semantics* θ*-security.*

**Definition** **18 (Semantic Type Sensitivity).** *Semantic type sensitivity indicates how sensitive a user is to a semantic type, and is calculated as shown in Equation (19), where* $n_{i}$ *indicates the number of times user* i *accesses semantic type* $S_{T}$ *, and* N *indicates the total number of times* $S_{T}$ *is accessed.*

(19) $$Sen(S_{T})=\frac{n_{i}}{N}$$

**Definition** **19. Semantic Popularity (SCP).** *Semantic Popularity indicates the hotness of a location, and the metric uses the idea of information entropy. Semantic Popularity in this section calculates the popularity of a semantic type as:*

(20) $$pop(S_{T})=2^{H(S_{T})}$$

*where* $H(S_{T})$ *is calculated as:*

(21) $$H(S_{T})=-\sum_{i=1}^{m}\frac{n_{i}}{N}\log\frac{n_{i}}{N}$$

*where* m *indicates the number of users who have accessed the semantic type.*

**Definition** **20. Trajectory Sensitive Rate (TSR).** *The trajectory sensitivity rate represents the sensitivity of a semantic location along a trajectory to the user and is expressed as*TSR*. Given a trajectory, the user’s trajectory sensitivity rate for that trajectory can be expressed as the sum of the user’s sensitivity value for the semantic type in that trajectory over the sum of all semantic prevalence, and the sensitivity value for the semantic type is the semantic type sensitivity*  $Sen(S_{T})$ *multiplied by the semantic prevalence* $pop(S_{T})$*. The semantic* TSR *is calculated as shown in Equation (22).*

(22) $$TSR(ST)=\frac{\sum_{S_{Ti\in ST}}Sen(S_{Ti})\cdot pop(S_{Ti})}{\sum_{S_{Ti}\in ST}pop(S_{Ti})}$$

**Definition** **21. Cosine similarity.** *The cosine similarity is used to calculate the semantic type similarity between two semantic trajectories, and the number of all semantic types along the trajectory is counted and calculated in the form of a vector. The cosine similarity is used in the PDPSP algorithm to find a trajectory from the anonymous set* TAS *that is most similar to the user’s original trajectory and is calculated as shown in Equation (23).*

(23) $$cos(ST_{1},ST_{2})=\frac{\sum_{i=1}^{n}\left(cnt(ST_{1}^{i})\cdot cnt(ST_{2}^{i})\right)}{\sqrt{\sum_{i=1}^{n}{\left(cnt(ST_{1}^{i})\right)}^{2}}\cdot \sqrt{\sum_{i=1}^{n}{\left(cnt(ST_{2}^{i})\right)}^{2}}}$$

**Definition** **22. Privacy Request.** *Unlike Definition 11, the privacy requirements in this section are denoted by* PR(STN,TL,θ). STN *denotes the number of semantic types contained in a single trajectory;* TL *denotes the number of anonymous trajectories, and* θ *denotes the trajectory semantic security threshold.*

### 5.3. A DP Personalized Protection Approach for Semantic Trajectories

#### 5.3.1. Algorithm Description

Most of the current trajectory privacy protection methods adopt the method of anonymous set construction. However, these methods do not consider the semantic information of users and the different sensitivity of each user to the semantic location when constructing the anonymous trajectory set. Hence, the anonymous set composed in this way not only has the problem that the information loss rate is relatively large, leading to low data availability, but it also cannot personalize users’ privacy protection. Thus, the set is easily inferred by attackers through the real trajectory of users and through background knowledge inference attacks.

In order to better protect the user’s trajectory against inference attacks, this section proposes the PDPST algorithm, which can personalize the user’s trajectory with privacy requirement settings. The algorithm mainly protects users’ trajectories from the perspective of anonymity set construction and trajectory publishing. The main steps of the algorithm are as follows.

(1) Add the user’s original trajectory to the trajectory anonymization set TAS.
(2) Calculate the trajectory sensitivity TSR of the trajectories in the trajectory set and count the number of semantic types in the trajectories.
(3) If the trajectory sensitivity TSR is bigger than semantic trajectory security threshold PR(θ), and the number of semantic types is greater than PR(STN), the trajectory will be added to the anonymity set TAS, and if the conditions are not satisfied, continue to execute step (2).
(4) Determine whether the anonymous trajectory parameter PR(TL) is reached. If this condition is satisfied, then go to the next step, and if not then continue to execute steps (2) to (3).
(5) Calculate the cosine similarity between the user’s original trajectory and the trajectory in the anonymous trajectory set and return a trajectory with the greatest cosine similarity $ST_{Result}$.
(6) Count the number of semantic types in trajectory $ST_{Result}$ and calculate the user’s sensitivity to the semantic types along the trajectory.
(7) The number of visits to each semantic type in this trajectory is perturbed by adjusting the parameters of Gaussian noise by semantic type sensitivity.
(8) Return a trajectory $ST^{'}$ after adding noise $ST^{'}$.

The PDPST algorithm first constructs an anonymous set that meets the user’s privacy budget, then selects a trajectory that is optimal according to the exponential mechanism optimal trajectory selection algorithm, and finally introduces an adjustable Gaussian noise mechanism to add noise to the number of semantic type visits in the selected optimal trajectory according to the semantic type sensitivity. The pseudo-code for the PDPST algorithm is shown in Algorithm 3.

In this pseudo-code, step 1 initializes the trajectory anonymity set and cosine similarity; step 2 adds the user’s trajectory to the trajectory anonymity set; steps 3 to 10 are for constructing the privacy-compliant trajectory anonymity set; steps 11 to 15 are for computing the optimal trajectory; and steps 16 to 29 are for the adjustable Gaussian noise addition process for each semantic type access frequency of the optimal trajectory.
**Algorithm 3.** PDPST algorithm pseudo-code.**Input: Original semantic trajectory of users**ST**, Track dataset**STS**, Privacy Needs**PR(STN,TL,θ) **Output: Semantic trajectory after adding privacy protection**$ST^{'}$**, Frequency of visits after two noise additions for semantic types**$\left(y_{1},y_{2}\right)$1. Initialize variables: track anonymous setTAS={∅}, Maximum cosine similarity Result=0;2. Add user raw semantic tracks to track anonymization sets,TAS={ST}; 3. **for** each $ST_{i}\in STS$ 4. **while**
TAS(TN)<PR(TN) 5.  Calculate TSR and count $ST_{i}(TSN)$ according to Equation (22);6.  **if** $ST_{i}(TSN)>PR(TSN)$ **and**
TSR<PR(θ);7.    $TAS=TAS\cup ST_{i}$;8.   **end if**9.  **end while**10 **.end for**11. **for** each $ST_{i}\in TAS$12.  Calculate the cosine similarity with the user’s real trajectory according to Equation (23): $cos(ST,ST_{i})$;13.  $Result=max(Result,cos(ST,ST_{i}))$;14. **end for**15. The corresponding trajectory with the highest cosine similarity is obtained according to Result: $ST_{{}_{Result}}$;16. $y_{1}=\{\emptyset \}$;17. **for** each $S_{Ti}\in ST_{Result}$18.  $count(S_{Ti})$;19.  Calculate the semantic type sensitivity of the user for semantic location type $S_{T_{i}}$ according to Equation (19): $Sen(S_{Ti})$;20.  $count{\left(S_{Ti}\right)}^{'}=count(S_{Ti})+v_{1}$;21.  $y_{1}=y_{1}\cup count{\left(S_{Ti}\right)}^{'}$; 22. **end for**23. $y_{2}=\{\emptyset \}$;24. **for** each $count{\left(S_{Ti}\right)}^{'}\in y_{1}$25. $\varepsilon_{2}=\varepsilon_{1}*Sen(S_{Ti})$,$\delta_{2}=\delta_{1}*Sen(S_{Ti})$;26.  $count{\left(S_{Ti}\right)}^{''}=count{\left(S_{Ti}\right)}^{'}+v_{2}$//Calculate the noise $V_{2}$ according to Equation (26) and select a sample point $v_{2}$ and add it to $count{\left(S_{Ti}\right)}^{'}$ 27.  $y_{2}=y_{2\cup }count{\left(S_{Ti}\right)}^{''}$;28. **end for**29. **return**
$ST^{'},(y_{1},y_{2})$

#### 5.3.2. Adjustable Gaussian Noise Mechanism

The Gaussian mechanism was introduced in Section 2 and is based on the Laplacian by setting a relaxation term δ with a probability δ of not satisfying strict DP, i.e., satisfying (ε,δ)-DP. This section combines the adjustable Gaussian mechanism in the literature [42] to dynamically adjust the semantic type sensitivity of the user in the trajectory to be published so that it guarantees the personalized semantic type privacy needs of the user. First of all, we add noise into the location to select the nearby semantics to build a candidate semantics set. Since different semantics have different importance to the user, we add noise to the semantics chosen in the set according to the different sensitivity of semantics, to realize the personalized protection.

Xiajie Du et al. [42] proposed a universal and quantifiable adjustable Gaussian mechanism, which is applicable to the case where the error parameter δ is not zero.

**Definition** **23 (Adjustable Gaussian privacy density function).** *If result* $y_{1}$ *obtained by adding Gaussian noise* $v_{1}\in V_{1}$ *to the current dataset* D *satisfies* $\left(\varepsilon_{1},\delta_{1}\right)$ *-DP, the privacy-preserving budget* $\varepsilon_{2}$ *is readjusted on this basis and noise* $v_{2}\in V_{2}$ *is added to obtain* $y_{2}$*. If we want to make* $\left(y_{1},y_{2}\right)$ *satisfy* $\left(\varepsilon_{2},\delta_{2}\right)$ *-DP, the probability distribution of adding noise* $V_{2}$ *in the case of noise* $V_{1}$ *is calculated as shown in Equation (24).*

(24) $$p(V_{2}=v_{2}|V_{1}=v_{1})=\frac{\varepsilon_{1}\sqrt{\ln(1.25/\delta_{1})}}{\varepsilon_{2}\sqrt{\ln(1.25/\delta_{2})}}e^{\frac{\varepsilon_{1}^{2}v_{1}^{2}}{4\ln(1.25/\delta_{1})}-\frac{\varepsilon_{2}^{2}v_{2}^{2}}{4\ln(1.25/\delta_{2})}}\Delta (v_{1}-v_{2})+\frac{\varepsilon_{2}^{2}-\varepsilon_{1}^{2}}{2\sqrt{\pi \ln(1.25/\delta_{2})}\varepsilon_{2}}e^{-\frac{\varepsilon_{2}^{2}v_{2}^{2}}{4\ln(1.25/\delta_{2})}}$$

Because the privacy budget $\varepsilon_{2}$ and privacy error $\delta_{2}$ of the second are obtained based on the product of the privacy budget $\varepsilon_{1}$ and privacy error $\delta_{1}$ of the first with the semantic type sensitivity, respectively, it is only necessary to prove that the result $\left(y_{1},y_{1}\right)$ after the privacy budget and privacy error of the two additions satisfies $\left(\varepsilon_{2},\delta_{2}\right)$-DP. The proof process is as follows.

Given two privacy budgets $\varepsilon_{1},\varepsilon_{2}$ and two privacy errors $\delta_{1},\delta_{2}$, $V_{1},V_{2}$ denoting the noise added twice, respectively, and given a dataset, $V_{1}$ satisfying $\left(\varepsilon_{1},\delta_{1}\right)$-DP, it follows from Definition 4 that the random function M satisfies $M=M+V_{1}$, and the PDF of $V_{1}^{}$ should be:

(25) $$p(V_{1}=v_{1})=\frac{\varepsilon_{1}}{2\sqrt{\pi \ln(1.25/\delta_{1})}}e^{-\frac{\varepsilon_{1}^{2}v_{1}^{2}}{4\ln(1.25/\delta_{1})}}$$

According to the conditional probability we get:

(26) $$p(V_{1}=v_{1},V_{2}=v_{2})=p(V_{1}=v_{1})p(V_{2}=v_{2}|V_{1}=v_{1})$$

According to Equations (24) and (25), the probability density function after noise is added can be calculated as:

(27) $$\begin{array}{l} p(V_{1}=v_{1},V_{2}=v_{2})=\frac{\varepsilon_{1}}{2\sqrt{\pi \ln(1.25/\delta_{1})}}e^{-\frac{\varepsilon_{1}^{2}v_{1}^{2}}{4\ln(1.25/\delta_{1})}}\times \\ \left(\frac{\varepsilon_{1}\sqrt{\ln(1.25/\delta_{1})}}{\varepsilon_{2}\sqrt{\ln(1.25/\delta_{2})}}e^{\frac{\varepsilon_{1}^{2}v_{1}^{2}}{4\ln(1.25/\delta_{1})}-\frac{\varepsilon_{2}^{2}v_{2}^{2}}{4\ln(1.25/\delta_{2})}}\Delta (v_{1}-v_{2})+\frac{\varepsilon_{2}^{2}-\varepsilon_{1}^{2}}{2\sqrt{\pi \ln(1.25/\delta_{2})}\varepsilon_{2}}e^{-\frac{\varepsilon_{2}^{2}v_{2}^{2}}{4\ln(1.25/\delta_{2})}}\right)\\ =\frac{\varepsilon_{1}^{2}}{2\sqrt{\pi \ln(1.25/\delta_{2})\varepsilon_{2}}}e^{-\frac{\varepsilon_{2}^{2}v_{2}^{2}}{4\ln(1.25/\delta_{2})}}+\frac{\varepsilon_{1}(\varepsilon_{2}^{2}-\varepsilon_{1}^{2})}{4\pi \sqrt{\ln(1.25/\delta_{1})}\sqrt{\ln(1.25/\delta_{2})}\varepsilon_{2}}e^{\frac{\varepsilon_{1}^{2}v_{1}^{2}}{4\ln(1.25/\delta_{2})}-\frac{\varepsilon_{2}^{2}v_{2}^{2}}{4\ln(1.25/\delta_{2})}} \end{array}$$

From the above equation, when the privacy budget and privacy error are reconciled for the second time, let the dataset $D_{1}$ strictly conform to DP $D_{2}$ for the part that does not conform to strict DP with privacy error probability $\delta_{2}$, $D=D_{1}+D_{2}$. Then Equation (27) can be obtained as:

$$p(M\in D)=P(M\in D_{1})+P(M(D_{2}))\leq e^{\varepsilon_{2}}p(M\in D_{1})+\delta_{2}$$

According to Definition 4, Equation (27) satisfies the DP Gaussian mechanism, i.e., the result $\left(y_{1},y_{2}\right)$ after adding noise twice satisfies $\left(\varepsilon_{2},\delta_{2}\right)$-DP.

#### 5.3.3. Algorithm Analysis

The PDPST algorithm proposed in this section first sets the privacy budget according to the user’s preference and constructs a trajectory anonymization set that conforms to the privacy budget; it then calculates the cosine similarity between the trajectory in the trajectory anonymization set and the real trajectory based on the semantic type vector to obtain a trajectory that is most similar to the user’s real trajectory. By counting the access frequency of each semantic type on this trajectory, it adds Gaussian noise that conforms to (ε,δ)-DP twice to the frequency of each semantic type to obtain $y_{1}$ and $y_{2}$, respectively, and proves that $\left(y_{1},y_{2}\right)$ conforms to $\left(\varepsilon_{2},\delta_{2}\right)$-DP. The PDPST algorithm can not only personalize the user’s track privacy, but also resist continuous query attacks by setting semantic track level parameters and semantic type parameters, tracking semantic θ-security, and perturbing the frequency of semantic type access.

### 5.4. Experiment and Analysis

#### 5.4.1. Experiment Setting

(1) Environment

This experiment uses a simulation environment with a Windows 10 operating system and the Python language for implementation, and the hardware environment is an Intel i5 4500u CPU, 16 GB RAM.

(2) Experimental Data

**Figure 11:**
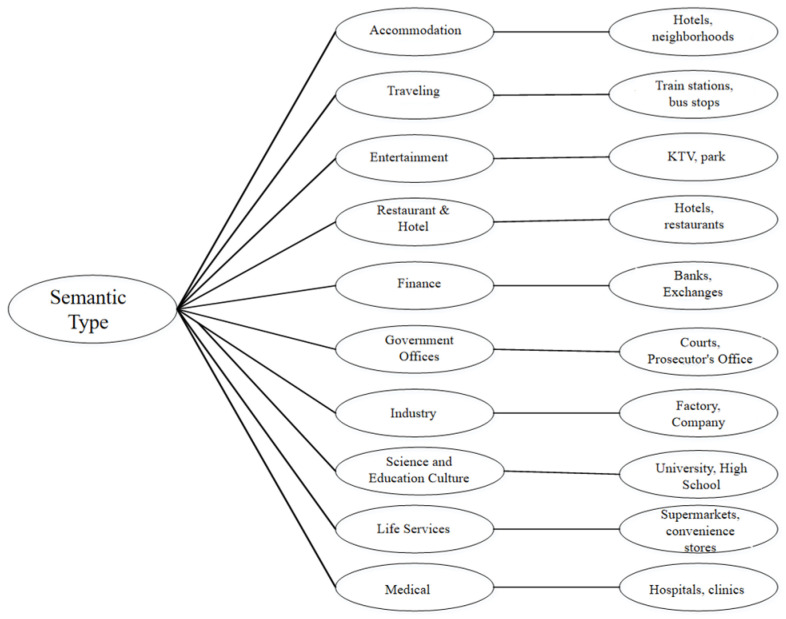
Semantic type classification.

The experimental data for this algorithm are taken from the Geolife [42,43] dataset, which includes 17,621 trajectories of 182 users from 2007 to 2012. This dataset contains a series of points in chronological order, each of which contains latitude and longitude information. These data record not only the location trajectories of users at home and in the workplace, but also the trajectories of a large range of outdoor activities, such as traveling, shopping, and cycling. The experiments in this section capture the semantic types corresponding to the location points on the map by calling the Baidu Map API interface along the original trajectory and classifying these semantic types into ten semantic types, as shown in Figure 11 below.

(3) Experimental parameters

In the experiment for the user’s privacy requirements, three parameters are included: the trajectory anonymity set parameter, the semantic type level parameter, and the trajectory semantics-security parameter. The experimental parameters of this experiment are shown in Table 1.

#### 5.4.2. Experimental Indicators

The PDPST algorithm proposed in this section is validated in terms of privacy protection degree, data availability, and running time.

(1) Privacy protection degree

Root mean squared error (RMSE) and mean absolute error (MAE) measure the data availability of the trajectory after protection is added. However, the security of the user’s trajectory should be considered along with the data availability. The trajectory similarity refers to the similarity between the selected optimal trajectory and the user’s original trajectory and is a performance indicator to measure the degree of privacy protection. The higher the trajectory similarity, the lower the probability that the attacker infers the user’s original trajectory information. In this section, the trajectory similarity is calculated using the cosine similarity of Equation (25) by transforming the semantic type kinds along the trajectory into vectors.

(2) Data Availability

To verify the data availability of the PDPST algorithm, MAE and RMSE are used as error indicators, and the smaller the error, the higher the data availability. The MAE and RMSE are calculated as follows:

(28) $$MAE=\frac{\sum_{1}^{N}\left|x_{i}-x\right|}{N}$$

(29) $$RMSE=\sqrt{\frac{1}{N}\sum_{i=1}^{N}{\left(x_{i}-x\right)}^{2}}$$

where $x_{i}$ denotes the data after adding noise, and x denotes the original data.

The anonymity success rate is also an important indicator of data availability. In this algorithm, the anonymity success rate is calculated as the number of semantic types in the user’s original trajectory that are under construction.

(3) Running time

In this algorithm, the running time consists of the anonymous time, optimal trajectory selection, and time for adding noise. For comparison with other variable control and conditional consistency algorithms, the anonymous time is used as the running time. In addition, the time consumption is calculated based on the semantic trajectory θ-change in security values and semantic type variables.

#### 5.4.3. Experimental Analysis

(1) Privacy protection degree

**Figure 12:**
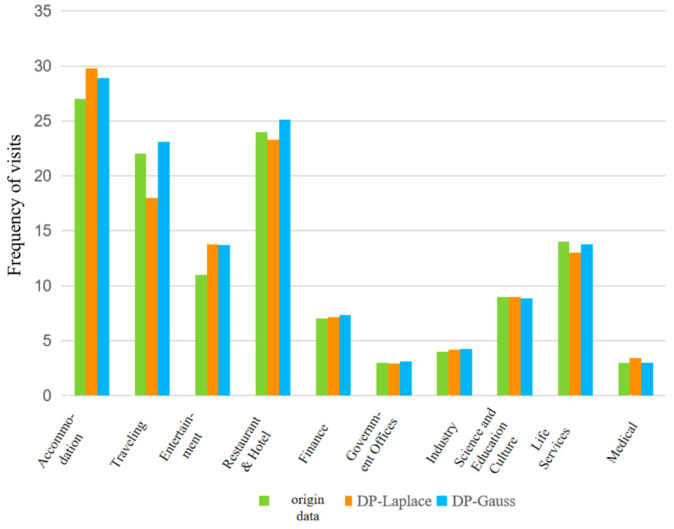
Comparison before and after noise is added to the original data.

The experiments in this section introduce the tunable Gaussian mechanism to the optimal trajectory selected from the anonymous trajectory set and add the tunable Gaussian noise and the tunable Laplace noise proposed in the literature [44] to the frequency of visits of each semantic type in the trajectory according to the calculated sensitivity of the semantic type. The comparison of the results obtained is shown in Figure 12.

As can be seen from Figure 12, the overall frequency of visits after adding adjustable Gaussian noise is closer than that of the original data. This is because, when adding adjustable Gaussian noise, the user’s sensitivity to each semantic type is considered. Finally, the privacy budget set according to the semantic type sensitivity is in line with the user’s privacy needs, so it is closer to the user’s original data, making it less easy for attackers to infer the original data and better protecting the user’s privacy.

**Figure 13:**
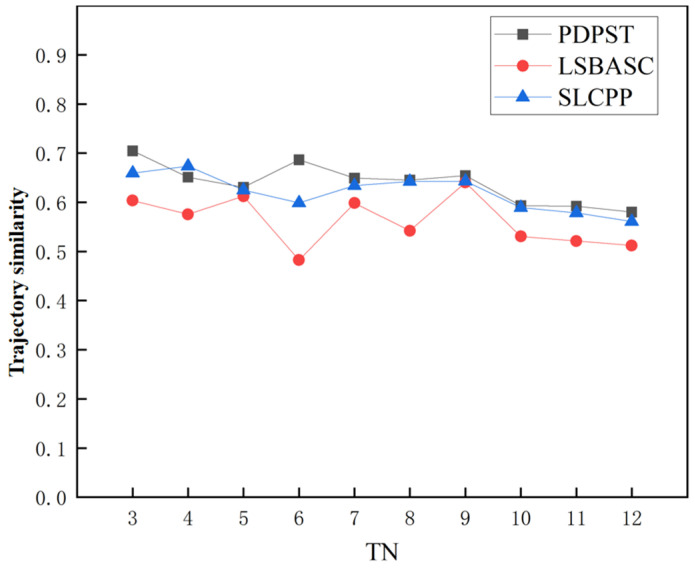
Trajectory similarity.

As shown in Figure 13, the SLCPP algorithm in the literature [45] and the LSBASC algorithm in the literature [46] were compared for trajectory similarity. It can be seen from the figure that the trajectory similarity of the PDPST algorithm improves by 0.02 on average compared to the SLCPP algorithm and by 0.1 on average compared with the LSBASC algorithm. The PDPST algorithm calculates the similarity between two trajectories using cosine similarity by constructing vectors for the semantic types along the trajectories, considering the similarity between the semantics.

(2) Data Availability

**Figure 14:**
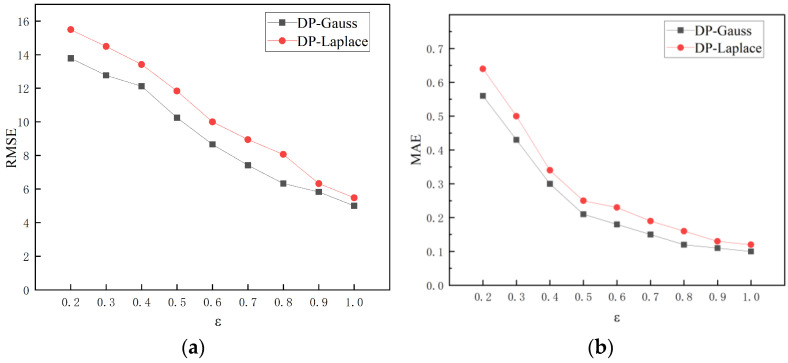
Error comparison chart: (**a**) root mean square error; (**b**) average absolute error.

There are two main measures of error in data availability: one is the root mean square error, as shown in Figure 14a, and the other is the mean absolute error, as shown in Figure 14b. As can be seen from the figure, the root means square error of both noise mechanisms decreases as the privacy budget increases but, on the whole, the adjustable Gaussian mechanism is smoother. If a new semantic type needs to be added, we will first search the nearby semantics, build a candidate semantics set, and select one of them as the published semantics.

**Figure 15:**
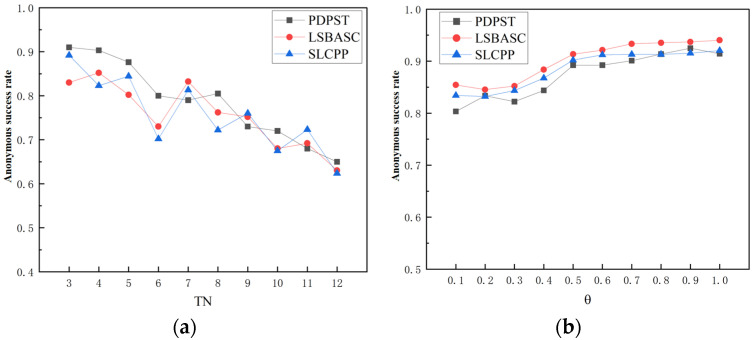
Anonymous success rate: (**a**) variation in anonymity success rate with (ε= 0.5); (**b**) variation in anonymity success rate with (TN = 10).

As can be seen in Figure 15a, when the trajectory semantic security threshold θ is taken to be 0.5, the anonymization success rate of the three algorithms decreases as the anonymization set K increases, but the overall fluctuation of the PDPST algorithm is smaller. Figure 15b shows the change in the anonymization success rate as the semantic security threshold θ increases when the anonymization set requirement K = 10. It can be seen from the figure that the success rate of all three algorithms increases with the increase in the semantic security threshold, and the increase in the anonymity success rate becomes larger when the value of θ changes from 0.4 to 0.5. The overall anonymity success rate of all three algorithms tends to be stable when θ is greater than 0.5. Because the PDPST algorithm considers the semantic parameter, the overall anonymity success rate is slightly lower than that of the other two algorithms, but the average anonymity success rate can reach higher than 0.85.

(3) Running time

**Figure 16:**
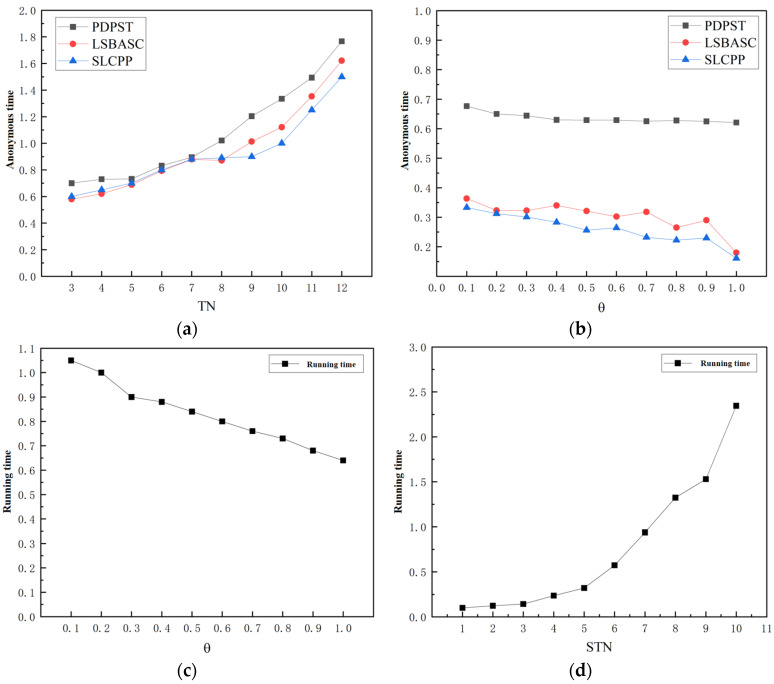
Runtime: (**a**) variation in anonymity time with (ε = 0.5); (**b**) variation in anonymity time with (ε = 10); (**c**) variation in running time with (ε = 5); (**d**) variation in running time with (ε = 0.5).

Figure 16a,b represents the comparison graphs of anonymization time for the three different algorithms. From Figure 16a, it can be seen that the anonymization time tends to increase as the number of trajectories in the anonymization set increases when the value of trajectory semantic safety θ is 0.5. The overall anonymization time of the LSBASC algorithm and SLCPP algorithm is lower than that of the PDPST algorithm because the PDPST algorithm needs to calculate the semantic types in the statistical trajectories when performing anonymization. As can be seen in Figure 16b, when the anonymization set TN is 10, the anonymization time of the three algorithms proceeds to shorten as the value of θ increases, which is because the more significant the value of θ, the less security required for the trajectory. The time spent for anonymization is shorter.

Figure 16c shows that when the number of trajectory semantic types is five and the number of anonymous trajectories TN is the default value, the overall running time of the PDPST algorithm varies with the value of θ. It can be seen that as the value of θ increases, the running time becomes shorter and shorter, and when the value of θ is 1, the overall time spent is the shortest. Figure 16d shows the variation in running time with STN when both θ and TN values are default values. It can be seen that as STN increases, the overall time spent increases, which is because when anonymous set construction is performed, the semantic types in each trajectory are counted. If they do not satisfy STN for the new trajectory, semantic types must be counted again.

## 6. Conclusions and Future Work

The rapid development of LBSs also results in new challenges. To create pushes according to users’ preferences, LBS providers upload and share a large amount of collected users’ location information. However, the shared location data may involve some sensitive user information. This paper proposes two corresponding privacy protection methods for semantic attacks in single-point location request services and the trajectory privacy leakage problem in continuous request services.

(1) This paper proposes a DP protection method for a semantic location to address the semantic attack problem in single-point location requests. We first construct an anonymous set for a semantic location that meets the user’s privacy requirements. Then, we introduce an indexing mechanism for DP to select an optimal semantic location from the anonymous set of semantic areas instead of uploading the real location to the server.
(2) To address the privacy leakage problem when trajectories are continuously queried, this paper proposes a personalized DP protection method for semantic trajectories that first constructs an anonymous set of trajectories according to the users’ privacy requirements. We build a vector based on the semantic types of the trajectories in the anonymous set and calculate their similarity using cosine similarity to obtain a trajectory that is most similar to the original trajectory. Finally, we introduce an adjustable Gaussian mechanism to add noise that matches the sensitivity of the users’ semantic types to the access frequency of the semantic types in the optimal trajectory, to improve the security of trajectory release and reduce the probability of attackers inferring the users’ location.

Although these two methods protect users’ semantic information to a certain extent, they still need to be improved in future research.

(1) When protecting users’ trajectory privacy, there are limitations in using cosine similarity to calculate the similarity of two trajectories, and the trajectory similarity should be calculated from multiple dimensions. This could be achieved by using a semantic similarity metric for each semantic type of the trajectory points, setting different weights for different semantic classes, and conducting a comprehensive weighted fusion to obtain trajectory selections from high to low.
(2) The server conducts privacy preservation in a central setting. In this case, we assume the server is trusted. In reality, however, the server is always located in a company (e.g., the Baidu Map server), so we cannot ensure the server is trusted. In the distributed architecture, users only trust themselves. We should find a solution to perturb the location semantics of the clients, rather than the server. Regardless of the noise perturbation mode or noise scale, it differs significantly from the central setting. In addition, the methods used to calculate the privacy budget (strength of privacy protection) in centralized and distributed approaches are also very different. A combination with localized differential privacy (LDP) is a potential solution that can be tried. However, there are still many problems in the distributed architecture, and we intend to continue to address these in the future.

## Figures and Tables

**Table 1:** Experimental parameter settings.

Parameters	Default Value	Range
Number of traces (Geolife)	17,612	
TN	10	3–12
STN	5	1–10
θ	0.5	0.1–1
Semantic Types	Accommodation, travel, entertainment hotels, finance, government agencies industry, science, education, life services medical	
Privacy Parameters ε	0.5	0.2–1
Privacy Error δ	0.0065	0.005–0.01

## Data Availability

Data is unavailable due to privacy or ethical restrictions.
